# The HPSE Gene Insulator—A Novel Regulatory Element That Affects Heparanase Expression, Stem Cell Mobilization, and the Risk of Acute Graft versus Host Disease

**DOI:** 10.3390/cells10102523

**Published:** 2021-09-23

**Authors:** Olga Ostrovsky, Polina Baryakh, Yan Morgulis, Margarita Mayorov, Nira Bloom, Katia Beider, Avichai Shimoni, Israel Vlodavsky, Arnon Nagler

**Affiliations:** 1 Chaim Sheba Medical Center, Department of Hematology and Bone Marrow Transplantation, Tel-Hashomer, Ramat Gan 5266202, Israel; Polina.Gidalevich@sheba.health.gov.il (P.B.); Yan.Morgulis@sheba.health.gov.il (Y.M.); Rita.Mayorov@sheba.health.gov.il (M.M.); Nira.Bloom@sheba.health.gov.il (N.B.); katiabeider@gmail.com (K.B.); avichai.shimoni@sheba.health.gov.il (A.S.); Arnon.Nagler@sheba.health.gov.il (A.N.); 2 Technion Integrated Cancer Center, Rappaport Faculty of Medicine, Technion, Haifa 3525433, Israel; vlodavsk@mail.huji.ac.il

**Keywords:** HPSE gene, enhancer, insulator, SNPs, allogeneic HSCT, acute GVHD

## Abstract

The HPSE gene encodes heparanase (HPSE), a key player in cancer, inflammation, and autoimmunity. We have previously identified a strong HPSE gene enhancer involved in self-regulation of heparanase by negative feedback exerted in a functional rs4693608 single-nucleotide polymorphism (SNP) dependent manner. In the present study, we analyzed the HPSE gene insulator region, located in intron 9 and containing rs4426765, rs28649799, and rs4364254 SNPs. Our results indicate that this region exhibits HPSE regulatory activity. SNP substitutions lead to modulation of a unique DNA-protein complex that affects insulator activity. Analysis of interactions between enhancer and insulator SNPs revealed that rs4693608 has a major effect on HPSE expression and the risk of post-transplantation acute graft versus host disease (GVHD). The C alleles of insulator SNPs rs4364254 and rs4426765 modify the activity of the HPSE enhancer, resulting in altered HPSE expression and increased risk of acute GVHD. Moreover, rs4426765 correlated with HPSE expression in activated mononuclear cells, as well as with CD3 levels and lymphocyte counts following G-CSF mobilization. rs4363084 and rs28649799 were found to be associated with CD34^+^ levels. Our study provides new insight into the mechanism of HPSE gene regulation and its impact on normal and pathological processes in the hematopoietic system.

## 1. Introduction

Mammalian cells express a single dominant functional heparanase (HPSE) that cleaves heparan sulfate (HS), contributes to disassembly of the extracellular matrix, releases HS-bound effector molecules, and thereby facilitates cancer metastasis and inflammation [1,2,3,4,5,6]. While human tissues reveal low expression of heparanase [5,6,7], constitutive or inducible heparanase expression was found in all hematopoietic cells, including neutrophils, activated lymphocytes, monocytes, macrophages, platelets, NK cells, mast cells, eosinophils, and dendritic cells [8,9,10]. A key role of heparanase in the function of the immune system has been identified [10].

Expression and activity of the HPSE gene are regulated by various transcription factors (e.g., SP1, EGR1), mechanisms (e.g., methylation), and gene regulators (e.g., estrogen, microRNAs, reactive oxygen species, glucose, fatty acids) at the DNA, RNA, and protein levels [11,12,13,14,15,16,17,18,19,20,21]. In a previous study, we identified a strong HPSE gene enhancer, located in intron 2 and involved in self-regulation of heparanase by negative feedback exerted in a functional rs4693608 SNP dependent manner [22].

Several functional single nucleotide polymorphisms (SNPs) of the HPSE gene have been identified [23,24,25], the most notable of which is rs4693608 polymorphism [9,25,26]. Combinations of rs4693608 and rs4364254 significantly correlate with heparanase levels in normal leukocytes and mononuclear cells (MNCs) in both peripheral and umbilical cord blood [9,25]. Importantly, a very significant association was observed between rs4693608 and the risk of acute and extensive chronic graft versus host disease (GVHD) post allogeneic stem cell transplantation (HSCT). Moreover, the mismatch between the recipient and donor in these SNPs significantly influences the risk of acute GVHD [9,26]. Notably, a positive association was found between recipients’ and donors’ rs4693608 discrepancy and the recovery time of neutrophils and platelets [9].

Genotype combinations (HR: high risk, MR: median risk and LR: low risk) of rs4693608 and rs4364254 SNPs were not randomly distributed. These combinations were grouped according to the first rs4693608 SNP, except for the AG-CC genotype. Heterozygous AG individuals for rs4693608, which possessed the rs4364254 CC genotype, exhibited low heparanase expression and a low risk of developing acute GVHD. However, the mechanism by which SNP rs4364254, located in intron 9, can alter the expression of HPSE remained to be elucidated [25,26,27].

ChIP-seq information from Roadmap Epigenomics, ENCODE and BLUEPRINT, which is publicly available in the Ensembl Database (http://apr2020.archive.ensembl.org/Homo_sapiens/Variation/Mappings?db=core;r=4:83301922-83302922;v=rs4426765;vdb=variation;vf=66317526 accessed on 20 July 2021), identified a 200 bp regulatory region (chr4: 83,302,401–83,302,600) with insulator activity in intron 9 of the HPSE gene. The CCCTC-binding factor (CTCF) binding site was identified at positions 83,302,472–83,302,476. The insulator-related CTCF is a key player in chromatin organization that regulates the formation of topologically associating domains and long-range chromatin loops in interphase nuclei [28]. CTCF is thought to be the primary essential of the insulator, a regulatory region that blocks the interaction between enhancers and promoters [28,29].

In the present study, we identified and analyzed an insulator region located in intron 9 of the HPSE gene and containing three polymorphic SNPs (rs4426765, rs28649799, and rs4364254). The present investigation was undertaken to analyze the interaction between the enhancer and insulator SNPs and the impact of these SNPs on the risk of developing acute GVHD after HSCT, as well as the extent of G-CSF-mediated peripheral blood stem cell mobilization. Our results reveal a new regulatory region in the HPSE gene, providing an insight into the regulation of heparanase gene expression and its effects on normal and pathological processes.

## 2. Materials and Methods

### 2.1. Study Population

The retrospective study included 674 consecutive patients (284 females and 390 males) with hematologic malignancies. The patients were transplanted at the Bone Marrow Transplantation Unit of the Chaim Sheba Medical Center (Tel Hashomer, Israel). All patients gave written informed consent, and the study was approved by the Ethics Committee of the Sheba Medical Center and the Israeli Ministry of Health (#4247). Patient characteristics are presented in Supplementary Table S1. Three hundred and sixty-five patients received grafts from human leukocyte antigen (HLA)-identical siblings, and 309 patients were transplanted from unrelated donors. The median age was 51 years (range 15 to 80 years). Conditioning regimens before HSCT, prophylaxis against GVHD, administration of granulocyte-colony stimulating factor (G-CSF), and prophylaxis with antibiotics and antimycotics were performed as previously described [9,26,30].

The effect of functional HPSE SNPs on G-CSF-mediated peripheral blood stem cell mobilization was analyzed in 280 consenting stem cell transplantation normal donors. Donors received G-CSF for 5 days. The daily dose of G-CSF was adjusted to donor weight (approximately 10 µg/kg/day), as recommended on the G-CSF label (480, 600, 780, or 960 µg/day) [31].

Total leucocytes from 108 unrelated healthy Israeli volunteers (53 females and 55 males) served as normal controls for analyzing the association of new insulator SNPs with heparanase mRNA expression levels. Their median age was 38 years (range 18 to 59 years). In addition, 104 peripheral blood (PB) samples from normal healthy individuals were examined for HPSE gene expression in untreated and LPS-treated mononuclear cells (MNCs). Median age was 39 years (range 25 to 56 years). All normal subjects gave their written informed consent and the study was approved by the Ethics Committee of the Sheba Medical Center and the Israeli Ministry of Health (#4247 and #2341-01-SMC). Human total leukocytes from healthy individuals and umbilical cord blood and primary cells from patients with T-ALL and B-ALL were applied for nuclear protein extractions. All subjects gave their written informed consent.

### 2.2. Cell Lines

Eight cell lines of various hematological malignancies (KG-1, MOLT-3, Jurkat, CEM, SU-DHL4, RPMI8226, NALM6, and 018Z) and 5 solid tumor cell lines (HT-1080, H1229, PC-3, MCF7, and PANC-1) were analyzed. Cell lines KG-1, MOLT-3, Jurkat, CEM, NALM6, and 018Z were kindly provided by Prof. Shai Izraeli (Chaim Sheba Medical Center, Tel-Hashomer, Israel) and were originally obtained from ATCC. Other cell lines were purchased from ATCC. The cells were maintained in log-phase growth in DMEM and RPMI1640 media (Gibco by Life Technologies, Inc., Paisley, UK) supplemented with 10% heat-inactivated fetal bovine serum (FBS), 100 U/mL penicillin, 1 mM L-glutamine, and 0.01 mg/mL streptomycin (Biological Industries, Beit HaEmek, Israel) in a humidified atmosphere with 5% CO_2_ at 37 °C. All cell lines were authenticated by STR DNA fingerprinting using the AmpFISTR Identifier Kit (Applied Biosystem Foster City, CA, USA).

### 2.3. SNPs Analysis

The genotypes of rs4693084 and rs4364254 SNPs were obtained by allele-specific amplification. Polymerase chain reaction (PCR) fragments were amplified from genomic DNA (Wizard^®^ Genomic DNA Purification kit, Promega, Madison, WI, USA). PCR reactions were performed as previously described [22]. Genotypes of rs4693608, rs4426765, and rs28649799 SNPs were identified using Real-Time SNP Assay. Custom-specific primers and probes were purchased from Bio Search Technologies (Novato, CA, USA). The PCR reaction mixture consisted of 5 µL of AccuStart Genotype Tough Mix Rox (Quanta, Gaithersburg, MD, USA) and 0.25 µL of primers and probes mix according to the manufacturer’s instructions. Reactions were performed using an ABI PRISM 7700 sequence detector (Applied Biosystems, Warrington, UK).

### 2.4. DNA Constructs

**Figure 1:**
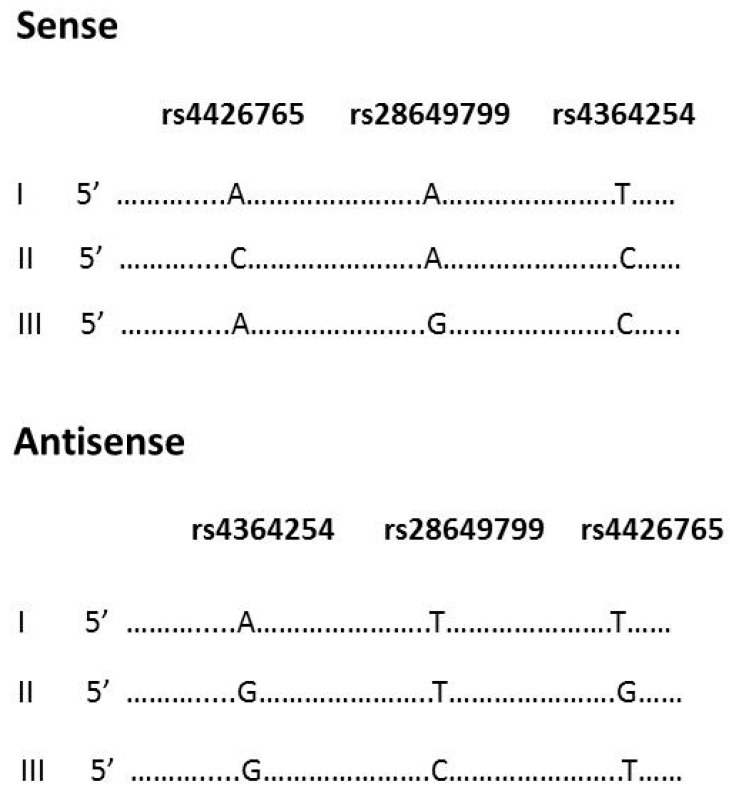
Structure of DNA constructs for luciferase reporter assay. Cloning of the HPSE gene fragment of intron 9 was performed using PCR II-TOPO vector. The insulator fragments were digested with *Hind* III and *Xho* I with subsequent ligation into phosphatase-treated pGL4.26 (luc2/minP/Hygro) vector. Three sense and three antisense DNA constructs were prepared. The constructs were designated with Roman numerals I, II, and III, and represent possible allelic variants in the general population.

A fragment of intron 9 (223 bp), which includes SNPs rs4426765, rs28649799, and rs4364254, was cloned by PCR via the PCR II-TOPO vector (Invitrogen by Life Technologies, Carlsbad, CA, USA) using the forward and reverse primers (Supplementary Table S2). The insulator fragments were digested with *Hind* III and *Xho* I and ligated into the phosphatase-treated pGL4.26 (luc2/minP/Hygro) vector (Promega, Madison, WI, USA) with subsequent cloning into JM109 Competent cells (Promega, Madison, WI, USA). Three sense and three antisense DNA constructs were prepared (Figure 1) followed by conformation of their direction by DNA sequencing.

### 2.5. Luciferase Reporter Assay

HT-1080, H1229, MCF7, PC3, RPMI8226, SU-DHL4, KG-1, Jurkat, CEM, NALM6, and MOLT3 cells were selected for Luciferase assay, performed in six-well or 12-well culture dishes according to the manufacturer’s instructions (AMAXA biosystems, Lonza, Germany). Cells were transfected using the Ingenio Electroporation Kit (Mirus Bio, Madison, WI, USA) and Nucleofector^TM^ (AMAXA biosystems, Lonza, Germany). Two µg of DNA construct were used for each electroporation. Twenty-four hours after reaction transfection, the cells were lysed in Passive Lysis Buffer (Promega, Madison, WI, USA). Firefly luciferase activity was measured by The Luciferase assay system (Promega, Madison, WI, USA) and 20/20^n^ Luminometer (Turner BioSystems, Sunnyvale, CA, USA). For normalization, total protein was quantified by the Bradford method (Bio-Rad, Hercules, CA, USA). Luciferase activity of pGL4.26 without insert was normalized to be 100%. All experiments were performed in triplicates and analyzed using a *t*-test. A *p* value ≤ 0.05 was calculated as statistically significant.

### 2.6. Electromobility Shift Assay (EMSA)

The primers applied to create allele-specific biotin-labeled probes are presented in Table S2. Method for generation of oligonucleotide probes was previously published [22]. The altered bases are marked in bold. Nuclear protein extracts were prepared using a Nuclear Extraction Kit (Millipore, Temecula, CA, USA). The Gelshift Chemiluminescent EMSA Kit (Active Motif, Rixensart, Belgium) was used according to the manufacturer’s protocol. EMSA reactions were performed as previously described [22].

### 2.7. Statistical Analysis

Genotype and allele frequencies of the SNPs were calculated by direct counting. All individuals were distributed into three groups according to three possible genotypes for each SNP. The means of mRNA relative quantification was calculated for each group. The Aspin–Welch unequal variances *t*-test was used to assess the association between the HPSE gene SNPs and the relative HPSE mRNA in normal total leukocytes and MNCs treated with LPS. This test is more reliable when the two groups have unequal variances and/or unequal sizes. G-CSF-mediated peripheral blood stem cell mobilization as a continuous variable, small groups with a low frequency of the corresponding alleles, and lack of the ability to suggest a normal distribution of variables allowed us to choose the Mann–Whitney U test for the statistical analysis. Acute GVHD was graded using the Glucksberg criteria [32]. The cumulative incidence of acute GVHD was calculated for both enhancer and insulator HPSE SNPs. Statistical analysis was carried out for grades II–IV acute GVHD. In the analysis of the cumulative incidence of GVHD, relapse was considered a competing risk [33]. Time to clinical event was measured from the date of HSCT. An analysis of the disparity between patient and donor was performed. A *p* value ≤ 0.05 was considered statistically significant. The NCSS 2007 software (NCSS, Kaysville, Utah, USA) was used for statistical analysis.

## 3. Results

### 3.1. Detection of Insulator Activity in Cancer Cell Lines of Hematological and Non-Hematological Origin

The ChIP-seq data, published in the Ensembl Database, identified a 200 bp regulatory region in the 9th intron of the HPSE gene (http://apr2020.archive.ensembl.org/Homo_sapiens/Variation/Mappings?db=core;r=4:83301922-83302922;v=rs4426765;vdb=variation;vf=66317526 accessed on 20 July 2021). The region was defined as an insulator according to the binding site of the CCCTC-binding factor (CTCF). The substitution of C to A for SNP rs4426765 disrupts the binding site of this transcription factor. Two additional polymorphic SNPs rs4364254 and rs28649799 are located in this region and can also alter the regulatory activity of the insulator.

To determine the presence of an active insulator in intron 9 and to study the functional effects of the three polymorphic SNPs, we used a luciferase reporter gene with a minimal promoter and measured luciferase activity. Cell lines derived from seven hematological malignancies (RPMI8226, SU-DHL4, KG-1, Jurkat, CEM, NALM6, and MOLT3) and four solid tumors (HT-1080, H1229, PC-3, and MCF7) were transiently transfected with each of the 6 DNA constructs or empty vector (Figure 1). Our results show that a 200 bp fragment of the HPSE gene exhibits regulatory activity in both the sense and antisense directions (Table 1). Of note, in most of the tested cell lines, the relative activity of luciferase was higher in constructs with an antisense direction of the insulator. Moreover, the constructs that included the allele A-A-T yielded increased levels of luciferase activity compared to the other constructs (Table 1). Also, the relative activity of luciferase was significantly higher in the solid tumor (especially HT1080), multiple myeloma (RPMI8226), and lymphoma (SU-DHL4) cells compared with low activity in the leukemic cell lines, particularly the CEM and MOLT3 acute lymphocytic leukemia cells. The effect of rs4426765 and rs4364254 SNPs on luciferase activity was evident in solid tumors and some of the hematological cancer cell lines (Table 1). Specifically, allele C of both SNPs significantly suppressed luciferase activity in the antisense direction.

### 3.2. Effect of rs4426765, rs28649799, and rs4364254 SNPs on the Binding of Nuclear Proteins to the Insulator Region

Nuclear extracts of hematological and solid tumor cell lines (NALM6, 018Z, H1229, and PANC-1), primary leucocytes from healthy adults and umbilical cord blood, as well as primary cells from patients with T-ALL and B-ALL were incubated with biotin-labeled probes (Supplementary Table S2) and subjected to electrophoretic mobility shift assay. Each probe included a corresponding SNP alteration. The DNA-protein complexes appeared as a band shift pattern. The specificity of the protein-oligonucleotide interaction was determined as previously described [22].

**Figure 2:**
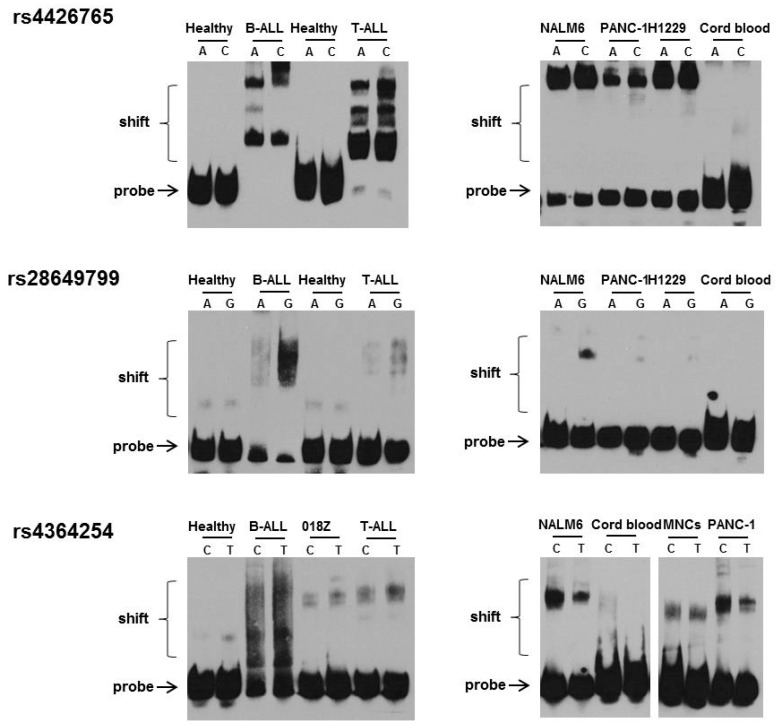
Electromobility shift assay (EMSA) of rs4426765, rs28649799, and rs4364254 SNPs using allele-specific oligonucleotide probes. Nuclear protein extracts from healthy donors, hematological malignancies and solid tumor cell lines, were incubated with three allele-specific biotin-labeled probes and analyzed by electrophoretic mobility shift assay. The order of the SNPs (rs4426765, rs28649799, and rs4364254) corresponds to their location in the insulator. In normal leukocytes, gel shift bands were allocated for both allelic probes rs28649799 and rs4364254 SNPs. In cord blood samples, nuclear-protein complexes were formed only with the C alleles of rs4426765 and rs4364254 SNPs. In the malignant cell lines, the band representing DNA-protein complexes shifted to a higher extent than in normal samples. The binding of protein complexes was found predominantly with the C, G, and C alleles of each SNP. Analysis of B-ALL and T-ALL primary samples exhibited the presence of large DNA/protein complexes formed by all three SNP probes. The affinity of the protein for the C allele of rs4426765 and the G allele of rs28649799 was higher compared to the A allele. The number of nuclear proteins bound to the rs4426765 probes was so large that the bands of unbound probes disappeared. Less noticeable was the disappearance of unbound probes for rs28649799 and rs4364254 SNPs.

Analysis of normal leukocyte samples from healthy controls revealed gel shift bands for both allelic probes in two (rs28649799 and rs4364254) out of the three SNPs, while in samples from umbilical cord blood nuclear-protein complexes were formed only with the C alleles of rs4426765 and rs4364254 SNPs (Figure 2). Remarkably, in the analyzed malignant cell lines, the band of DNA-protein complex was shifted significantly more than in the normal cell samples (Figure 2). Furthermore, the formation of DNA/protein complexes was found predominantly with the C or G alleles of each SNP (Figure 2).

EMSA analysis of samples from patients with B-ALL and T-ALL exhibited the presence of large DNA/protein complexes formed by all three SNP probes (Figure 2). Each probe disclosed its own pattern. The affinity of the protein for the C allele of rs4426765 and the G allele of rs28649799 was higher compared to the A allele, respectively. As seen in Figure 2, the number of nuclear proteins that were associated with the rs4426765 probe was so large that it led to disappearance of the unbound probes. Additionally, the affinity of the T-ALL patient samples for DNA was lower compared to that of B-ALL cells. Less noticeable was the disappearance of unbound probes rs28649799 and rs4364254. These results indicate that the highest amount of nuclear proteins bind to the insulator region which contains the CTCF binding site.

### 3.3. Acute GVHD

To assess the role of the HPSE insulator in predicting the risk of acute GVHD post-HSCT, two patient groups from our previous studies [9,26] were pooled and analyzed. Three hundred and ten patients developed acute GVHD, 53 (17.1%) grade I, 125 (40.3%)—grade II, 49 (15.8%)—grade III, and 83 (26.7%)—grade IV, while 261 did not.

The univariate analysis of the cumulative incidence of clinically significant acute GVHD (grades II–IV) in association with two enhancers (rs4693608, rs4693084) and three insulators (rs4426765, rs28649799, and rs4364254) SNPs and their combinations, is presented in Table 2. rs4693608 is the main SNP associated with the risk of acute GVHD. The cumulative incidence of acute GVHD on day 100 (grade II–IV) was 47.9% (95% CI 41.2–55.5) for recipients carrying the AA genotype, 39.4% (95% CI 34.2–45.4) for recipients with the AG genotype, and 29.1% (95% CI 22.4–37.8) for recipients with the GG genotype (*p* = 0.000419, Table 2). Additional two SNPs (rs4693084 and rs4364254) tended to be correlated with the risk of acute GVHD (*p* = 0.067 and *p* = 0.076, respectively, Table 2).

We subsequently analyzed the interaction between enhancer (rs4693608) and insulator (rs4364254 and rs4426765) SNPs. For this, the cumulative incidence of acute GVHD was calculated for each genotype separately (Table 3). This approach disclosed that the rs4364254 CC genotype reduces the risk of acute GVHD in patients with heterozygous rs4693608 AG genotype (20.7%; 95% CI 10.2–42.2). An opposite effect was obtained for the AA-TC insulator genotype. This SNP combination dictates an increased risk of acute GVHD in both rs4693608 AG (55.2%; 95% CI 42.0–72.4) and GG (46.7%; 95% CI 30.2–72.0) carriers, respectively (Table 3). The low frequency of certain genotypes (AA-CC-TT, AA-CC-TC, AA-AA-CC, and AA-CC-CC) in the general population did not allow assessing the risk of acute GVHD in these patients. We, therefore, combined them into one group that demonstrated a high risk of acute GVHD—66.7% (95% CI 46.6–95.4) (Table 3).

In our previous studies [25,26] the combination of rs4693608 and rs4364254 SNPs was evaluated. This approach allowed distribution of all possible HPSE genotype combinations into three groups (HR, MR, and LR) correlating with high, intermediate, and low heparanase mRNA expression levels and high, intermediate and low risk of acute GVHD, respectively. In the present study, the cumulative rate of acute GVHD incidence (grade II–IV) for recipients of group HR was 47.1% (95% CI 40.4–55.1), recipients of group MR was 41.1% (95% CI 35.5–47.5), and for recipients of group LR was 27.6% (95% CI 21.6–35.3) on day 100 post-HSCT (*p* = 0.000121, Table 2).

Based on enhancer-insulator interaction results (Table 3), updated SNP groups were allocated. The N-HR group includes all individuals with the rs4693608 AA genotype and AG-AA-TC recipients from the AG heterozygote group. The N-MR group consists of carriers AG-AC-TC, AG-CC-TC, AG-NN-TT, and GG-AA-TC, and the N-LR group includes the genotypes GG-AC-TC, GG-NN-TT, GG-NN-CC, and AG-NN-CC. NN is any genotype (AA, AC, or CC) of rs4426765 SNP. The cumulative incidence of day 100 grade II–IV acute GVHD was 49.2% (95% CI 43.1–56.0) for N-HR recipients, 39.6% (95% CI 33.9–46.4) for N-MR, and 22.5% (95% CI 16.5–30.7) in the N-LR group, respectively (*p* < 0.00001, Table 2).

We previously reported that disparities between recipient and donor pairs in SNP combinations of the HPSE gene significantly increase the likelihood of developing acute GVHD post-HSCT [9,26]. All recipient-donor pairs were divided into three groups according to the potential risk for acute GVHD development. The first cohort, D1, contained pairs with high risk for developing acute GVHD (HR-MR and HR-LR pairs). The second group, D2, consisted of pairs with moderate risk of acute GVHD development (HR-HR, MR-MR, MR-HR, and MR-LR). The third group, D3, included three pairs with a low risk of developing acute GVHD (LR-LR, LR-MR, and LR-HR pairs) [26]. Now we performed a univariate analysis of the cumulative incidence of clinically significant acute GVHD in the renewed recipient-donor pairs (Table 2). Cumulative incidence of acute GVHD was 56.9% (95% CI 47.6–68.1) in group D1, 42.1% (95% CI 36.8–48.0) in group D2 and 23.2% (95% CI 17.1–31.5) in group D3 (*p* < 0.00001) (Table 2).

### 3.4. Correlation between Enhancer and Insulator HPSE SNPs and Heparanase mRNA Levels in MNCs before and after LPS Treatment

Applying activated PB MNCs, we have shown statistically significant differences between HPSE expression levels and rs4693608, rs4364254, and their genotype combinations among healthy individuals [9]. HPSE expression in steady-state MNCs showed little or no differences among individuals with various HPSE gene genotypes (Table 4). In contrast, analysis of the correlation between new insulator SNPs (rs4426765 and rs28649799) and HPSE gene expression in LPS treated MNCs revealed a very significant association with rs4426765. The level of HPSE expression was relatively high in carriers of the AA and AC genotypes and relatively low in carriers of the CC genotype (Table 4). The ratio test revealed a strong correlation between rs4693608, rs4426765, and rs4364254 SNP genotypes and the increase in HPSE expression levels in response to LPS stimulation. Healthy people with genotypes AA, AA, and TT increased HPSE levels to a greater extent than their counterparts with genotypes GG, CC, and CC, respectively (Table 4).

Next, we investigated how interactions between the three significant SNPs (rs4693608, rs4426765, and rs4364254) affect HPSE expression in LPS stimulated MNCs. First, the interaction between the two insulator SNPs (rs4426765 and rs4364254) was analyzed. We allocated all individuals into three groups depending on the level of HPSE expression. Group 1 included genotypes AA-TT and AA-TC, group 2 included AC-TT and AC-TC, and group 3 the CC-TC, CC-CC, AC-CC, and AA-CC genotypes. Comparison of heparanase expression between AA-TT and AA-TC (group 1), among AC-TT and AC-TC (group 2), and between AC-CC and CC-CC (group 3) did not reveal any differences (Table 5), whereas highly significant differences were observed between the three groups (Table 5). Group 1 exhibited a higher level of HPSE mRNA compared to the other two groups (*p* = 0.025), while group 3 expressed a low level of HPSE (*p* = 0.0029). Subsequently, the interaction between the enhancer and insulator SNPs was studied. It became clear that the best way to understand how insulator SNPs alter enhancer activity is to analyze heterozygous individuals with intermediate HPSE expression (Table 5). Heterozygous AG individuals with genotypes AA-TT and AA-TC in the insulator disclosed relatively high levels of HPSE expression, while heterozygous individuals with two or more C alleles in the insulator SNPs exhibited relatively low levels of HPSE gene expression (*p* = 0.023, Table 5). Thus, we concluded that an active insulator with C alleles decreases the activity of the HPSE gene enhancer and thereby diminishes HPSE expression levels. Of note, the same analysis of homozygous individuals with genotypes AA and GG for rs4693608 did not reveal any difference in the correlation analysis (Table 5).

These results are consistent with the enhancer-insulator interaction found in the group of patients with acute GVHD (Table 3). Recipients with the AG-AA-TC genotype had a higher cumulative incidence risk, while those with the AG-NN-CC genotype revealed a lower risk of acute GVHD (Table 3). Association analysis between the updated groups of enhancer-insulator and the relative expression of HPSE mRNA in LPS treated MNCs disclosed that individuals with the N-LR genotype displayed low levels of heparanase compared to those with the N-MR and N-HR genotypes (*p* = 0.0041) (Table 4).

### 3.5. Association between Enhancer and Insulator HPSE Gene SNPs and the Levels of HPSE mRNA in Normal Leukocytes

Our previous study showed that HPSE rs4693608 and rs4364254 SNPs correlated with HPSE mRNA expression in total leucocytes [25]. Subsequent investigations have suggested that this correlation is restricted to neutrophils [9]. In the present study, new rs4426765 and rs28649799 insulator SNPs were analyzed for correlation with HPSE mRNA levels in total leukocytes among healthy individuals. In addition, the interaction between enhancer and insulator SNPs was examined. Healthy individuals with genotypes GG (rs4693608) or CC (rs4364254) disclosed relatively low levels of HPSE (*p* = 0.0028 and 0.00049, respectively), while individuals with genotypes AA (rs4693608) or TT (rs4364254) expressed relatively high levels of HPSE mRNA. In contrast, the new insulator SNPs (rs44267765 and rs28649799) disclosed no correlation (Table 6). Previous genotype combination groups HR, MR, and LR are presented in Table 6. Correlation analysis between the updated groups of enhancer-insulator SNPs and the relative expression of HPSE mRNA in normal total leucocytes was performed. Individuals with the N-LR genotype combination exhibited low levels of heparanase compared to those with the N-MR and N-HR genotypes (*p* = 0.000076) (Table 6). This association revealed better results compared to previous rs4693608—rs4364254 SNP combinations (*p* = 0.000076 for the N-LR group vs. *p* = 0.00098 for the LR group compared to the other groups, respectively, Table 6). The results indicate that the complicated interaction between rs4693608 enhancer SNP and rs44267765 and rs4364254 insulator SNPs significantly determine heparanase expression variability among normal persons.

### 3.6. G-CSF-Mediated Peripheral Blood Stem Cell Mobilization

G-CSF-mobilized peripheral blood stem/progenitor CD34^+^ cells are the main graft source used for HSCT [34]. We have previously shown that G-CSF treatment resulted in high-affinity binding of heparanase to the enhancer region of the HPSE gene, followed by increased heparanase expression in donor cells [22,27].

Two hundred and ten donors (106 females and 174 males) were included in the analyses. The median age was 45 (range, 19–73) years. All the observed polymorphisms were in the Hardy–Weinberg equilibrium. The parameters of mobilization included in the analyses are presented in Table 7. One enhancer SNP (rs4363084) and one insulator SNP (rs28649799) were found to correlate with CD34^+^ (Table 7). The overall CD34^+^ yield, as well as CD34^+^ × 10^6^/kg, were higher in carriers of the TT genotype compared to those with GG and GT genotypes (*p* = 0.0068 and *p* = 0.025, respectively). The insulator SNP rs28649799 also revealed an association with the low-frequency G allele (*p* = 0.039). Analysis of the interaction between these two SNPs indicated that only TT-AA, GG-AG, and TT-AG disclosed an increased number of CD34^+^ cells, whereas the GT-AG genotype showed a lower level, similar to that of other genotype combinations. The TT-AG genotype exhibited the highest CD34^+^ mobilization yield. The additive effect of both SNPs may explain this result. However, the frequency of genotypes TT-AA, GG-AG, and TT-AG in the general population is low. Therefore, additional studies applying large population cohorts are needed to clarify the function of both SNPs in G-CSF mediated mobilization.

In addition, the insulator rs4426765 SNP was found to correlate with CD3 cell numbers following G-CSF administration. Notably, cell numbers were found to be higher in carriers of the CC genotype compared to carriers of the AA and AC genotypes (*p* = 0.044 for total yield, *p* = 0.039 for CD3^+^ × 10^8^/kg, and *p* = 0.0026 for % CD3, respectively) (Table 7). In addition, leukocyte counts after mobilization were higher in donors with CC genotypes compared to donors with other genotypes (21.5 × 10^3^/µL, median range 10.3–29.0 for carriers of CC genotype vs. 9.95 × 10^3^/µL, median range 8.4–12.2, and 10.05 × 10^3^/µL, median range 7.5–12.2 for possessors of AA and AC genotypes, respectively, *p* = 0.013) (Table 7). Some trend to correlation was observed between insulator rs4426765 SNPs and level of platelets after G-CSF administration (*p* = 0.059). The number of thrombocytes was higher in the possessors of CC genotype (375.6 × 10^3^/µL, median range 304.7–485.7) compared to donors with AA (323.9 × 10^3^/µL, median range 300.2–349.8) and AC (311.9 × 10^3^/µL, median range 269.8–349.0) genotypes (Table 7).

## 4. Discussion

Even though noncoding regions constitute over 98% of the human genome, epigenetic profiling studies have shown that more than 80% of the human genome is potentially functional [35]. Insulators are classes of DNA sequence elements that have a common ability to protect genes from inappropriate signals proceeding from their surrounding environment [36]. An insulator element may act as an enhancer-blocker by disrupting enhancer-promoter interactions, when positioned between enhancer and promoter regions, without rendering the enhancer inactive. Insulators can also protect genes by acting as “barriers” that prevent the advance of nearby condensed chromatin that may otherwise silence gene expression. While barrier activity prevents transcriptional repression, its enhancer-blocking property interferes with transcriptional activation [36].

In the present study, we identified a new regulatory region in intron 9 of the HPSE gene. According to ChIP-seq data, the CCCTC-binding factor binds to this region. In mammals, a zinc-finger protein, CTCF, binds to most of the known insulator sequences [36], suggesting that the newly identified 200 bp regulatory region is an insulator. There are three polymorphic SNPs in this region of the HPSE gene. C to A substitution in SNP rs4426765 destroys the CTCF binding site. Analysis of the luciferase reporter revealed cellular- and SNP-dependent regulatory activity in this region. EMSA disclosed binding of the DNA/protein complex to both alleles with a higher affinity for allele C. Importantly, all the examined malignant cell lines and primary leukemia samples disclosed a more prominent shift of the main bands compared to normal cell samples. This result is likely due to the formation of DNA/protein complexes with additional proteins that were bound to the probes. Binding of protein complexes was found mainly to the C or G alleles of rs4426765, rs28649799, and rs4364254.

While subsets of CTCF binding sites are known to be associated with insulators, many insulators can function independently of CTCF [29]. Correlation analyses disclosed that HPSE insulator function is not dictated solely by binding of the CTCF transcription factor. Our results suggest that additional proteins are involved in the formation of a DNA-protein complex in the insulator region and that insulator SNPs can alter the composition and affinity of these complexes.

**Figure 3:**
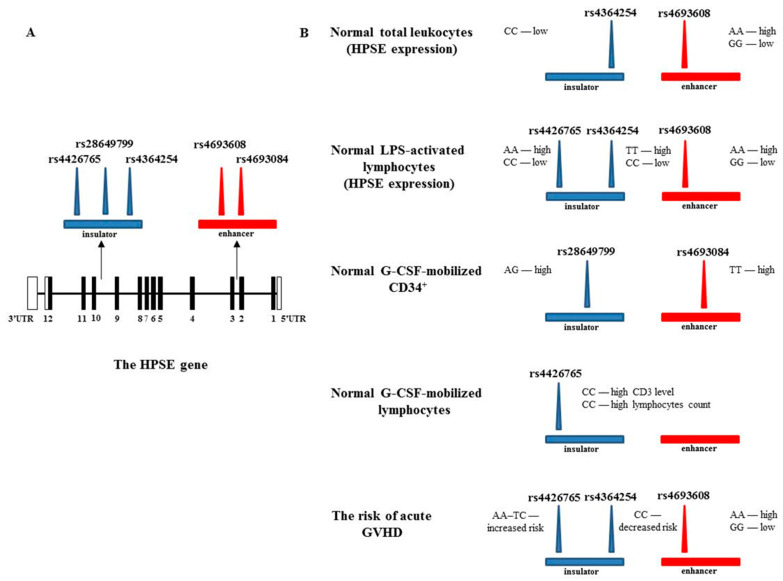
Scheme describing the correlation between enhancer and insulator SNPs with heparanase expression, G-CSF mobilization, and the risk of acute GVHD after HSCT. (**A**). HPSE gene map. The numbered boxes represent 12 exons. Shaded rectangles signify an open reading frame, and open boxes indicate the 5′- and 3′-untranslated regions. Arrows mark the location of the enhancer (red rectangle) and insulator (blue rectangle). The three insulator SNPs and two enhancer SNPs analyzed are marked as blue and red triangles, respectively. (**B**). Impact of heparanase SNPs on various processes.

Figure 3 presents a summary diagram describing the identified significant correlations between enhancer and insulator SNPs with heparanase expression, G-CSF mobilization, and the risk of acute GVHD after HSCT. Location of the enhancer (red rectangle) and insulator (blue rectangle) is indicated relative to the HPSE gene map (Figure 3A) and the effect of HPSE SNPs on various processes is shown in Figure 3B.

Correlation analysis of HPSE enhancer and insulator SNPs in different groups of healthy individuals and transplanted patients revealed that rs4364254 and rs4426765 play an important role in the insulator activity. We have previously described a significant correlation between rs4364254 and progesterone receptor (PR) expression in breast cancer patients [27]. High expression of the progesterone receptor correlated with the C allele, and low expression with the T allele (*p* = 0.002) [27]. PR-positive breast cancers exhibit improved prognosis [37,38]. In addition, the C allele of rs4364254 SNP was found to be associated with a low risk of developing Veno occlusive disease of the liver [39]. These results suggest that the CC genotype of the rs4364254 insulator SNP decreases the enhancer activity and thereby diminishes the expression level of the HPSE gene and possibly its protumorigenic and proinflammatory activities.

Our results emphasize the role of the CTCF-associated rs4426465 in lymphocyte activity and mobilization after G-CSF treatment. Carriers of the CC genotype disclosed a low level of HPSE expression following incubation with LPS in comparison to carriers of the AC and AA genotypes, differences that are highly significant. In addition, significantly improved G-CSF induced CD3 levels and higher lymphocyte counts were found in CC genotype donors. These results are complementary to our previously published findings, in which we analyzed the effect of LPS on the ability of DNA/protein complexes to bind the strong intron 2 enhancer of the HPSE gene [22]. Exposure to LPS resulted in the disappearance of DNA/protein complexes in normal MNCs and decreased the affinity of DNA/protein complexes in U937 monocytic cells [22]. In contrast, exposure to G-CSF caused high-affinity interaction of nuclear proteins with the enhancer region, associated with increased HPSE expression in donor cells [22,27]. It is conceivable that the HPSE intron insulator strongly regulates the function of the enhancer and thereby affects heparanase involvement in lymphocyte activation and function. Additional confirmation supporting the importance of rs4426465 SNP in lymphocyte function was provided by the EMSA results in B- and T-ALL patient samples. A large number of nuclear proteins bind to the insulator region containing the CTCF binding site. The affinity of nuclear proteins for allele C was higher than that of allele A. The size of DNA/protein complexes decreased upon the progressive diverging from SNP rs4426465 to distal polymorphisms along the insulator nucleotide sequence.

The enhancer SNP rs4693608 exhibited a leading function in the expression of the HPSE gene in normal neutrophils and LPS-activated MNCs, as well as in predicting the risk of acute GVHD post-transplantation. Analysis of the interaction between enhancer and insulator SNPs (especially in heterozygous individuals for the enhancer rs4363608) provides insight into the mechanism by which an insulator can alter enhancer activity. Genotype CC of rs4364254 SNP reduces the activity of the HPSE gene enhancer. Individuals with the AG-CC genotype were found to have low levels of HPSE expression in normal neutrophils and LPS-activated MNCs, and a low risk of developing acute GVHD. In contrast, the AA-TC genotype from rs4426765 and rs4364254 insulator SNPs increased the level of HPSE expression in LPS-activated MNCs. Moreover, the AA-TC insulator genotype increased the risk of acute GVHD in carriers of the AG and GG enhancer genotypes.

Heparanase is known to play a decisive role in tumor metastasis and angiogenesis [5]. High expression of heparanase is frequently observed in a long list of primary human tumors of various etiologies, in correlation with high vessel density and a poor clinical outcome [40,41,42,43]. The enzyme is also involved in inflammation [44], fibrosis [45], diabetes [46], and kidney dysfunction [47]. The enhancer and insulator of the HPSE gene may be involved in the development of these and other pathological processes. It is important to note; however, that the interaction between enhancer and insulator can vary depending on the nature of cells and the heparanase-related processes.

Heparanase-inhibiting compounds, antibodies, and small molecules are being developed [48,49,50]. Four different heparin mimics were subjected to clinical trials in cancer patients. However, none of the molecules reached the stage of approval for clinical use. Elucidation of the precise mechanism(s) of HPSE gene regulation will facilitate rational development of heparanase-specific inhibitors.

It is worth noting that over the past several years, new and potential technologies have been developed (e.g., nuclease-deficient zinc fingers, TALEs, and CRISPR fusion) with the potential of treating diseases by modulating gene expression, an approach termed cis-regulation therapy (CRT) [51]. These approaches can be used to modify the activity of gene-regulatory elements such as promoters, enhancers, silencers, and insulators, thereby modulating the expression levels of their target genes aiming at achieving therapeutic effects. Future development of such technologies will hopefully allow fine-tuning of heparanase expression for the treatment of diseases associated with dysregulation of HPSE gene expression.

## Figures and Tables

**Table 1:** Effect of SNPs on the reporter activity of the HPSE gene insulator.

Cell Lines	Relative Luciferase Activity			*p* Value	Relative Luciferase Activity			*p* Value
	Sense (%)			A-A-T/C-A-C	Antisense (%)			A-A-T/C-A-C
				A-A-T /A-G-C				A-A-T /A-G-C
	A-A-T	C-A-C	A-G-C	C-A-C/A-G-C	A-A-T	C-A-C	A-G-C	C-A-C/A-G-C
Solid tumors:								
HT1080	455	552	590	<0.01; <0.01; 0.051	2188	1471	2226	<0.01; NS; <0.01
H1229	134	131	182	NS; <0.01; <0.01	261	184	181	<0.01; <0.01; NS
PC3	189	219	167	0.021; 0.054; <0.01	355	273	229	<0.01; <0.01; 0.023
MCF7	159	113	160	<0.01; NS; <0.01	259	138	117	<0.01; <0.01; NS
Hematological malignancies:								
RPMI8226	154	101	206	<0.01; <0.01; <0.01	220	225	134	NS; <0.01; <0.01
SU-DHL4	199	125	167	<0.01; 0.17; <0.01	400	211	146	<0.01; <0.01; <0.01
KG1	100	115	164	NS; <0.01; <0.01	129	160	224	0.019; <0.01; <0.01
Jurkat	164	124	72	<0.01; <0.01; <0.01	102	77	90	0.038; NS; NS
CEM	56	56	72	NS; NS; NS	101	59	73	<0.01; <0.01; 0.051
NALM6	97	79	160	0.013; <0.01; <0.01	115	138	117	0.048; NS; NS
MOLT3	87	78	89	NS; NS; NS	86	85	54	NS; <0.01; <0.01

NS: non-significant.

**Table 2:** Univariate analysis of cumulative incidence of clinically significant (II–IV) acute GVHD in association with patient enhancer and insulator HPSE gene SNPs.

SNPs	Genotype	Cumulative Incidence, (95% CI), %	χ^2^, *p* Value
**rs4693608** (enhancer)	AA	47.9 (41.2–55.5)	**15.55**
	AG	39.4 (34.2–45.4)	
	GG	29.1 (22.4–37.8)	**0.00042**
**rs4693084** (enhancer)	GG	42.0 (37.2–47.4)	5.4
	GT	38.8 (32.3–46.5)	
	TT	17.5 (7.9–38.9)	0.067
**rs4426765** (insulator)	AA	42.2 (37.1–47.9)	2.97
	AC	37.1 (31.1–44.1)	
	CC	34.6 (23.5–50.9)	0.23
**rs28649799** (insulator)	AA	38.5 (34.4–43.1)	3.78
	AG	42.8 (34.2–53.6)	
	GG	60.0 (29.3–100)	0.15
**rs4364254** (insulator)	TT	39.3 (34.1–45.4)	5.16
	TC	43.7 (37.9–50.4)	
	CC	28.5 (20.3–39.9)	0.076
**rs4693608**	HR	47.1 (40.4–55.1)	**18.04**
**rs4364254**	MR	41.1 (35.5–47.5)	
	LR	27.6 (21.6–35.3)	**0.00012**
**rs4693608**	N-HR	49.2 (43.1–56.0)	**30.67**
**rs4426765**	N-MR	39.6 (33.9–46.4)	
**rs4364254**	N-LR	22.5 (16.5–30.7)	**<0.00001**
**Discrepancy**	D1: N-HR/N-MR	56.9 (47.6–68.1)	**33.67**
	N-HR/N-LR		
	D2: N-HR/N-HR	42.1 (36.8–48.0)	**<0.00001**
	N-MR/N-MR		
	N-MR/N-HR		
	N-MR/N-LR		
	D3: N-LR/N-LR	23.2 (17.1–31.5)	
	N-LR/N-MR		
	N-LR/N-HR		

Significant deviations (*p* < 0.05) are marked in bold. HR: AA-TT and AA-TC genotype combinations; MR: AG-TT and AG-TC genotype combinations; LR: GG-TT, GG-TC, GG-CC, and AG-CC genotype combinations. N-HR: AA-AA-TT, AA-AA-TC, AA-AC-TT, AA-AC-TC, AA-NN-NN, and AG-AA-TC genotype combinations (NN is any SNP genotype of rs4426765 and rs4364254 SNPs). N-MR: AG-AA-TT, AG-AC-TT, AG-AC-TC, AG-CC-TC, and GG-AA-TC genotype combinations. N-LR: GG-AA-TT, GG-AC-TT, GG-AC-TC, GG-AA-CC, GG-AC-CC, GG-CC-CC, and AG-NN-CC genotype combinations (NN is any SNP genotype of rs4426765).

**Table 3:** Univariate analysis of cumulative incidence of clinically significant (II-IV) acute GVHD: examination of interaction between patient rs4693608, rs4426765, and rs4364254 SNPs.

rs4693608-rs4426765-rs4364254	Cumulative Incidence, (95% CI), %
AA-AA-TT	45.5 (37.2–55.6)
AA-AA-TC	44.4 (21.4–92.3)
AA-AC-TT	50.0 (28.4–88.0)
AA-AC-TC	45.8 (31.6–66.6)
**AA-NN-NN**	**66.7 (46.6–95.4)**
AG-AA-TT	39.0 (30.3–50.2)
**AG-AA-TC**	**55.2 (42.0–72.4)**
AG-AC-TT	36.8 (20.5–66.4)
AG-AC-TC	39.0 (29.9–50.7)
AG-CC-TC	40.4 (21.6–75.6)
**AG-NN-CC**	**20.7 (10.2–42.2)**
GG-AA-TT	18.8 (8.5–41.5)
**GG-AA-TC**	**46.7 (30.2–72.0)**
GG-AC-TT	16.7 (2.8–99.7)
GG-AC-TC	29.2 (15.6–54.4)
GG-AA-CC	25.0 (7.5–83.0)
GG-AC-CC	32.9 (15.9–68.1)
GG-CC-CC	23.4 (10.8–50.2)

The genotype AA-NN-NN includes AA-CC-TT, AA-CC-TC, AA-AA-CC, AA-AC-CC, and AA-CC-CC genotype combinations. The genotype AG-NN-CC contains AG-AA-CC, AG-AC-CC, and AG-CC-CC genotype combination.

**Table 4:** Association between enhancer and insulator HPSE gene SNPs and heparanase mRNA expression levels in MNCs from healthy individuals before and after LPS treatment.

SNPs	Treatment	Genotype	No.	mRNA Level	Comparisons to Carriers	*p* Value
				(AV ± SE)		
rs4693608 (enhancer)	Pre-treatment	AA	33	11.3 ± 3.1	AA to GG	0.123
		AG	53	9.5 ± 1.3	AA to others	0.25
	Post-treatment	GG	23	6.3 ± 1.1	GG to others	**0.028**
		AA		47.3 ± 15.6	AA to GG	**0.033**
	Ratio	AG		41.8 ± 10	AA to others	0.4
		GG		12.2 ± 2.5	GG to others	**0.00054**
		AA		10.5 ± 4.1	AA to GG	**0.047**
		AG		5.6 ± 1.3	AA to others	0.16
		GG		2.0 ± 0.25	GG to others	**0.0029**
rs4426765 (insulator)	Pre-treatment	AA	60	10.2 ± 1.7	AA to CC	0.43
		AC	35	7.7 ± 1.7	AA to others	0.28
	Post-treatment	CC	8	7.7 ± 2.5	CC to others	0.59
		AA		48.4 ± 11.7	AA to CC	**0.0078**
	Ratio	AC		21.7 ± 5.3	AA to others	**0.027**
		CC		14.0 ± 4.5	CC to others	**0.0078**
		AA		8.8 ± 2.5	AA to CC	**0.0059**
		AC		3.5 ± 0.6	AA to others	**0.026**
		CC		1.8 ± 0.3	CC to others	**0.0019**
rs28649799 (insulator)	Pre-treatment	AA	83	8.9 ± 1.2	AA to AG	0.71
	Post-treatment	AG	19	10.6 ± 3.4		
	Ratio	AA		37.8 ± 8	AA to AG	0.72
		AG		32.4 ± 16.2		
		AA		5.7 ± 1.3	AA to AG	0.53
		AG		9.3 ± 5.1		
rs4364254 (insulator)	Pre-treatment	TT	47	9.4 ± 1.8	TT to CC	0.35
		TC	45	9.4 ± 1.9	TT to others	0.84
	Post-treatment	CC	12	6.9 ± 1.8	CC to others	0.29
		TT		50.2 ± 13.4	TT to CC	**0.012**
	Ratio	TC		27.6 ± 7.7	TT to others	0.089
		CC		14.2 ± 3.1	CC to others	**0.0039**
		TT		7.5 ± 2.3	TT to CC	**0.04**
		TC		6.1 ± 2.2	TT to others	0.45
		CC		2.6 ± 0.6	CC to others	**0.013**
rs4693608 & rs4364254 (enhancer & Insulator)	Pre-treatment	HR	31	11.6 ± 4.8	HR to LR	0.17
		MR	46	8.9 ± 1.3	HR to others	0.31
	Post-treatment	LR	27	6.7 ± 1.1	LR to others	0.087
		HR		46.8 ± 16.4	HR to LR	**0.047**
	Ratio	MR		43.5 ± 11.2	HR to others	0.42
		LR		12.5 ± 2.2	LR to others	**0.011**
		HR		10.5 ± 4.1	HR to LR	**0.049**
		MR		6.0 ± 1.4	HR to others	0.16
		LR		2.1 ± 0.3	LR to others	**0.0034**
rs4693608 & rs4364254 & rs4426765 (enhancer & Insulator)	Pre-treatment	N-HR	44	11.2 ± 2.4	N-HR to N-LR	0.16
		N-MR	38	7.8 ± 1.3	N-HR to others	0.23
	Post-treatment	N-LR	22	7.2 ± 1.3	N-LR to others	0.17
		N-HR		46.0 ± 13.1	N-HR to N-LR	**0.017**
	Ratio	N-MR		39.0 ± 11.7	N-HR to others	0.28
		N-LR		13.0 ± 2.3	N-LR to others	**0.0016**
		N-HR		9.2 ± 3.1	N-HR to N-LR	**0.028**
		N-MR		5.5 ± 1.5	N-HR to others	0.13
		N-LR		2.1 ± 0.3	N-LR to others	**0.0041**

AV—average; SE—standard error (SE = SD/√N, SD—standard deviation). Significant deviations (*p* < 0.05) are marked in bold.

**Table 5:** rs4693608, rs4426765, and rs4364254 interaction in healthy individuals after LPS treatment.

SNPs Interaction	Genotype	No.	mRNA Level (AV ± SE)	Comparisons to Carriers	*p* Value
rs4426765	Group 1:AA-TT	39	56.7 ± 16.0	AA-TT to AA-TC	0.3
rs4364254	AA-TC	19	33.4 ± 15.3	AC-TT to AC-TC	0.67
				AC-CC to CC-CC	0.79
into insulator	Group 2:AC-TT	7	19.2 ± 8.0		
	AC-TC	24	23.9 ± 7.3		
				Group1 to Group3	**0.0052**
	Group 3:CC-TC	1	7.3	Group1 to others	**0.025**
	CC-CC	7	15.0 ± 5.1	Group3 to others	**0.0029**
	AC-CC	4	13.2 ± 4.0		
	AA-CC	1	12.2		
rs4693608	AA-1	23	49.8 ± 20.6	AA-1 to AA-2	0.95
rs4426765	AA-2	5	47.5 ± 28.9		
rs4364254	AA-3	1	7.3		
enhancer-insulator	AG-1	29	56.7 ± 17.1	AG-1 to AG-3	**0.023**
	AG-2	17	21.1 ± 6.3	AG-1 to others	**0.047**
	AG-3	4	14.2 ± 5.0	AG-3 to others	**0.021**
	GG-1	6	9.2 ± 5.6	GG-1 to GG-3	0.49
	GG-2	9	12.4 ± 3.8	GG-1 to others	0.54
	GG-3	8	14.2 ± 4.2	GG-3 to others	0.57

Significant deviations (*p* < 0.05) are marked in bold.

**Table 6:** Association between enhancer and insulator HPSE gene SNPs and relative heparanase mRNA expression level among healthy individuals.

SNPs	Location	Genotype	No.	mRNA Level	Comparisons to Carriers	*p* Value
				(AV ± SE)		
rs4693608	Enhancer	AA	27	13.9 ± 2.3	AA to GG	**0.01**
		AG	62	11.7 ± 1.6	AA to others	0.2
		GG	19	7.2 ± 1.1	GG to others	**0.0028**
rs4426765	Insulator	AA	59	13 ± 1.7	AA to CC	0.56
		AC	36	9.3 ± 1.4	AA to others	0.14
		CC	12	11.3 ± 2.2	CC to others	0.91
rs28649799	Insulator	AA	84	11.4 ± 1.2	AA to AG	0.73
		AG	22	12.5 ± 2.8		
rs4364254	Insulator	TT	49	12.7 ± 1.8	TT to CC	**0.0029**
		TC	50	11.3 ± 1.5	TT to others	0.32
		CC	9	6.1 ± 1	CC to others	**0.00049**
rs4693608 & rs4364254	Enhancer & Insulator	HR	25	14.6 ± 2.4	HR to LR	**0.007**
		MR	59	11.9 ± 1.7	HR to others	0.16
		LR	22	7.1 ± 0.9	LR to others	**0.00098**
rs4693608 & rs4364254 & rs4426765	Enhancer & Insulator	N-HR	35	14. 7 ± 2.3	N-HR to N-LR	**0.0014**
		N-MR	55	11.1 ± 1.5	N-HR to others	0.082
		N-LR	16	6.4 ± 0.7	N-LR to others	**0.000076**

AV—average; SE—standard error (SE = SD/√N, SD—standard deviation). Significant deviations (*p* < 0.05) are marked in bold.

**Table 7:** Association analysis between enhancer and insulator HPSE gene SNPs and mobilization parameters after granulocyte colony-stimulating factor (G-CSF) administration to healthy donors.

Parameters	rs4693608			rs4693084			rs4426765			rs28649799		
	Genotype	Median (Range)	*p* *	Genotype	Median (Range)	*p* *	Genotype	Median (Range)	*p* *	Genotype	Median (Range)	*p*
CD34^+^ × 10^6^ total yield	AA (89)	753.9	0.12	GG (175)	691.9	**0.011**	AA (139)	659.7	0.54	AA (204)	656.7	0.34
		(631.7–812.5)			(620.6–768.0)			(549.0–755.4)			(549.4–729.4)	
	AG (125)	634	0.12	GT (78)	561	0.26	AC (90)	675.6	0.67	AG (41)	682.5	
		(545.9–711.3)			(479.1–651.4)			(521.3–758.9)			(495.8–988.7)	
	GG (61)	598.9	0.28	TT (8)	1022.3	**0.0068**	CC (15)	524.9	0.55			
		(493.1–727.1)			(598.9–1196.6)			(431.4–866.1)				
CD34^+^ × 10^6^/kg	AA (64)	8.9	0.82	GG (138)	7.9	**0.026**	AA (106)	7.9	0.85	AA (172)	7.7	**0.039**
		(7.0–10.7)			(7.2–9.1)			(7.0–9.4)			(7.3–9.1)	
	AG (105)	7.7	0.86	GT (66)	8.2	0.53	AC (82)	8.4	0.52	AG (32)	10.9	
		(7.4–9.1)			(7.5–9.5)			(7.3–10.7)			(7.5–13.1)	
	GG (53)	8.3	0.54	TT (7)	11.9	**0.025**	CC (15)	7.4	0.99			
		(6.9–10.0)			(7.6–23.5)			(5.5–11.9)				
% CD34^+^	AA (89)	0.84	0.29	GG (175)	0.75	0.22	AA (138)	0.75	0.9	AA (203)	0.72	**0.081**
		(0.69–0.92)			(0.7–0.84)			(0.65–0.83)			(0.66–0.77)	
	AG (126)	0.72	**0.08**	GT (78)	0.68	0.38	AC (91)	0.72	0.9	AG (42)	0.84	
		(0.62–0.77)			(0.57–0.76)			(0.66–0.77)			(0.69–1)	
	GG (59)	0.74	0.83	TT (8)	0.85	0.17	CC (15)	0.77	0.94			
		(0.64–0.84)			(0.49–1.29)			(0.59–0.85)				
CD3^+^ × 10^8^ total yield	AA (88)	270.7	0.99	GG (173)	276	0.73	AA (138)	273.7	**0.054**	AA (202)	275.2	0.41
		(255.4–300.0)			(262.8–300.2)			(258.3–297.8)			(264.6–300.0)	
	AG (124)	289.1	0.54	GT (78)	274.5	0.58	AC (89)	266.6	0.87	AG (41)	268.4	
		(266.6–307.5)			(258.3–305.4)			(247.0–298.9)			(222.4–306.9)	
	GG (61)	268.9	0.67	TT (8)	266.2	0.77	CC (15)	336	**0.044**			
		(241.3–299.2)			(187.1–425.5)			(270.6–378.1)				
CD3^+^ × 10^8^/kg	AA (64)	3.6	0.44	GG (137)	3.8	0.63	AA (105)	3.6	**0.039**	AA (171)	3.7	0.72
		(3.1–4.0)			(3.4–4.0)			(3.2–3.9)			(3.5–4.0)	
	AG (104)	3.9	0.69	GT (66)	3.8	0.83	AC (82)	3.9	0.37	AG (32)	3.8	
		(3.5–4.3)			(3.2–4.3)			(3.3–4.2)			(3.0–4.7)	
	GG (53)	3.8	0.33	TT (7)	4	0.61	CC (15)	4.16	**0.039**			
		(3.4–4.2)			(2.2–5.5)			(3.5–5.9)				
% CD3^+^	AA (88)	31.3	0.63	GG (174)	31.3	**0.04**	AA (138)	31.8	**0.0042**	AA (202)	31.4	0.97
		(28.9–33.9)			(29.6–32.4)			(30.3–33.6)			(29.8–32.7)	
	AG (126)	32.3	0.65	GT (78)	33.9	0.58	AC (90)	29.5	0.98	AG (42)	31.7	
		(29.6–34.0)			(30.6–36.9)			(27.7–32.0)			(28.1–34.3)	
	GG (59)	31.9	0.76	TT (8)	26	**0.03**	CC (15)	39.9	**0.0026**			
		(29.8–35.9)			(10.8–29.8)			(32.4–43.9)				
Neutrophils abs., 10^3^/µL	AA (90)	13.9	0.66	GG (175)	14.3	**0.032**	AA (140)	14.9	0.25	AA (205)	14.4	0.41
		(11.2–17.0)			(12.5–17.0)			(12.5–19.2)			(12.5–17.0)	
	AG (124)	15.6	0.37	GT (79)	14.1	0.45	AC (91)	16.1	0.56	AG (42)	17.1	
		(12.4–20.6)			(11.3–17.4)			(12.0–19.2)			(12.6–25.2)	
	GG (61)	13.6	0.91	TT (8)	24.8	**0.042**	CC (15)	13	0.25			
		(11.4–19.1)			(10.0–42.7)			(4.5–20.6)				
Neutrophils, %	AA (90)	43.4	0.77	GG (176)	41.5	0.12	AA (140)	43.1	**0.017**	AA (206)	40.6	0.54
		(37.5–49.5)			(35.7–47.3)			(37.3–47.6)			(33.2–44.8)	
	AG (126)	37	0.48	GT (80)	37.7	0.95	AC (92)	40.7	0.59	AG (42)	43.7	
		(31.6–46.9)			(29.2–44.3)			(31.9–52.6)			(32.1–55.0)	
	GG (61)	38.7	0.82	TT (8)	56.8	0.12	CC (15)	28.2	**0.015**			
		(31.2–53.3)			(33–71.7)			(12.5–44.3)				
Lymphocytes abs., 10^3^/µL	AA (90)	9.8	0.75	GG (175)	9.9	0.12	AA (140)	9.9	**0.017**	AA (205)	10	0.94
		(7.4–12.3)			(8.8–12.2)			(8.4–12.2)			(9.4–12.2)	
	AG (124)	10	0.57	GT (79)	10.8	0.95	AC (91)	10	0.64	AG (42)	11.2	
		(8.5–12.1)			(8.1–13.9)			(7.5–12.2)			(6.6–15.8)	
	GG (60)	10.3	0.99	TT (8)	3.7	0.1	CC (15)	21.5	**0.013**			
		(6.6–13.9)			(0.48–15.8)			(10.3–29.0)				
Lymphocytes, %	AA (90)	31.8	0.77	GG (176)	26.5	**0.049**	AA (140)	23.5	**0.026**	AA (206)	26.3	0.91
		(19.7–38.8)			(19.7–34.4)			(18.8–32.1)			(19.7–34.3)	
	AG (126)	22.4	0.66	GT (80)	26.4	0.83	AC (92)	25.6	0.54	AG (42)	24.4	
		(17.8–26.8)			(19.7–38.6)			(17.3–35.4)			(15.2–38.6)	
	GG (61)	30.4	0.47	TT (8)	9.5	**0.049**	CC (15)	55.9	**0.023**			
		(19.9–40.8)			(0.65–29.64)			(29.0–63.9)				
Platelets, 10^3^/µL	AA (89)	297.8	0.32	GG (175)	323.7	0.24	AA (138)	323.9	**0.068**	AA (204)	324	0.22
		(258.6–326.5)			(303.9–339.9)			(300.2–349.8)			(304.7–345.8)	
	AG (125)	349	**0.018**	GT (79)	324.7	0.29	AC (92)	311.9	0.98	AG (42)	327.9	
		(323.7–371.3)			(282.3–366.0)			(269.8–349.0)			(286.1–426.6)	
	GG (61)	324.7	0.72	TT (8)	369.5	0.29	CC (15)	375.6	**0.059**			
		(274.4–342.8)			(209.0–485.7)			(304.7–485.7)				

* The *p* value was indicated according to the following model: 1st *p* value—comparison between two homozygous genotypes; 2nd *p* value—correspondence of the first homozygous genotype to other genotypes; 3rd *p* value—comparison of the second homozygous genotype with other genotypes. Significant deviations (*p* ≤ 0.05) and the trend towards significance are marked in bold.

## Data Availability

Not applicable.

## Supplementary Materials

The following are available online at https://www.mdpi.com/article/10.3390/cells10102523/s1, Table S1: Characteristics of transplant recipients and donors; Table S2: Primer sequences for insulator cloning and generation of allele-specific probes.
