# Supplementary material for: The HPSE Gene Insulator—A Novel Regulatory Element That Affects Heparanase Expression, Stem Cell Mobilization, and the Risk of Acute Graft versus Host Disease
Source: Cells. 2021 Sep 23;10(10):2523. doi: 10.3390/cells10102523 (PMC8534152; doi:10.3390/cells10102523)
Supplement: Supplementary file 1 [file cells-10-02523-s001.zip › cells-1327851-supplementary.pdf]

cells

MDPI

Article

The HPSE Gene Insulator—A Novel Regulatory Element That Affects Heparanase Expression, Stem Cell Mobilization, and the Risk of Acute Graft versus Host Disease

Olga Ostrovsky [1,*], Polina Baryakh [1], Yan Morgulis [1], Margarita Mayorov [1], Nira Bloom [1], Katia Beider [1], Avichai Shimoni [1], Israel Vlodavsky [2] and Arnon Nagler [1]

Table S1. Characteristics of transplant recipients and donors.

Variable	Characteristic	No. of cases (%)
Age (years)	Median: 51	
	range: 15–80	
Gender	Recipient:	
	male	390 (57.9)
	female	284 (42.1)
	Donor:	
	male	413 (61.3)
	female	261 (38.7)
	Male/male	240 (35.6)
Recipient/Donor	Male/female	150 (22.3)
	Female/male	173 (25.7)
	Female/female	111 (16.5)
Diagnosis	AML	291 (43.2)
	ALL	86 (12.8)
	CML	33 (4.9)
	CLL	23 (3.4)
	MDS	69 (10.2)
	HL	17 (2.5)
	NHL	86 (12.8)
	MM	41 (6.1)
	MF	15 (2.2)
	AA	13 (1.9)
Donor	Sibling	365 (54.2)
	MUD/mm	309 (45.8)
Conditioning	Myeloablative	384 (57.0)
regimen	Reduced-intensity conditioning	290 (43.0)

Abbreviations: ALL, acute lymphoblastic leukemia; AML, acute myeloid leukemia; AA, aplastic anemia; CML, chronic myeloid leukemia; CLL, chronic lymphocytic leukemia; HL, Hodgkin's lymphoma; MDS, myelodysplastic syndrome; MF, myelofibrosis; MM, multiple myeloma; MUD/mm, matched unrelated or mismatched unrelated donor; NHL, Non-Hodgkin's lymphoma; M, male; F, female.

Table S2. Primer sequences for insulator cloning and generation of allele-specific probes

Name	Modification	Sequence of primers	PCR/probe product size, bp
Insulator cloning		forward 5' TGTTATCCTGGGGGCATTTA 3'	223
		reverse 5' TTGGCTTTGAGCTTTGCTTT 3'	
rs4426765 probe	A → C	A allele forward:	35
		5' [Btn]ATTCATCTTTTTTTTTTTTTTC**A**CCTCACATGTGA 3'	
		A allele reverse:	35
		5' [Btn]TCACATGTGAGG**T**GAAAAAAAAAAAAAAGATGAAT 3'	
		C allele forward:	
		5' [Btn]ATTCATCTTTTTTTTTTTTTTC**C**CCTCACATGTGA 3'	
		C allele reverse:	
		5' [Btn]TCACATGTGAGG**G**GAAAAAAAAAAAAAAGATGAAT 3'	
rs28649799 probe	A → G	A allele forward: 5' [Btn]CATAGCCTGTAGGGG**A**TATCTGTCAAGAATG 3'	31
		A allele reverse: 5' [Btn]CATTCTTGACAGATA**T**CCCCTACAGGCTATG-3'	
		G allele forward: 5' [Btn]CATAGCCTGTAGGGG**G**TATCTGTCAAGAATG 3'	31
		G allele reverse: 5' [Btn]CATTCTTGACAGATA**C**CCCCTACAGGCTATG 3'	
rs4364254 probe	T → C	T allele forward: 5' [Btn]AGTTTAAGTATTCTTGGTTAT**T**GTTCATCC 3'	30
		T allele reverse: 5' [Btn]GGATGAAC**A**ATAACCAAGAATACTTAAACT 3'	
		C allele forward: 5' [Btn]AGTTTAAGTATTCTTGGTTAT**C**GTTCATCC 3'	30
		C allele reverse: 5' [Btn]GGATGAAC**G**ATAACCAAGAATACTTAAACT 3'	
